# Sensitivity and Specificity of Bedside Qualitative Troponin I Test Kit as Compared with the Standardized Quantitative Lab Test for Troponin I

**DOI:** 10.7759/cureus.8201

**Published:** 2020-05-19

**Authors:** Syed Arsalan Ahmed, Javed Ismail, Taha Nafees, Ahmad Faraz, Muhammad Tahir, Mustaqueem Ur Rehman

**Affiliations:** 1 Medicine, Karachi Medical and Dental College, Karachi, PAK; 2 Cardiology, Western Sydney University, Sydney, AUS; 3 Internal Medicine, Karachi Medical and Dental College, Karachi, PAK; 4 Trauma and Orthopaedics, Leeds Teaching Hospitals NHS Trust, Leeds, GBR; 5 Orthopaedics, Jinnah Postgraduate Medical Center, Karachi, PAK; 6 Cardiology, Tabba Heart Institute, Karachi, PAK

**Keywords:** trop i, sensitivity, specificity, qualitative, quantitative, myocardial infarction

## Abstract

Background and objective

Myocardial infarction (MI) is the leading cause of mortality and morbidity worldwide. Early diagnosis of MI remains the mainstay of prompt treatment. Thus, the shorter the door-to-needle time, the more efficient is the emergency department (ED) to cope with a heart attack emergency. To improve the diagnosis of MI, this study aimed to determine the sensitivity and specificity of (qualitative) troponin I kit against the quantitative lab test for troponin I.

Materials and methods

Patients of both genders admitted at the Karachi Institute of Heart Diseases with acute coronary syndrome (ACS)/non-ST-elevation myocardial infarction (NSTEMI) were administered a standardized questionnaire. Quantitative analysis of troponin I was carried out by the hospital laboratory. The sample was simultaneously used for qualitative analysis of troponin I using the troponin I test kit.

Results

We recruited 200 patients comprising 134 (67%) males and 66 (33%) females. In total, 130 (65%) were hypertensive, 64 (32%) had dyslipidemia, 56 (28%) presented with a family history of MI, 60 (30%) had diabetes mellitus, 56 (28%) were smokers, and 24 (12%) presented with a previous history of MI. The kit showed 98% sensitivity and 100% specificity as compared to the quantitative test with a cutoff of 0.30 ng/dl, i.e., the quantitative test showed 128 positive and 72 negative cases, whereas the qualitative test showed 125 positive and 75 negative cases. The differences in test results were on values of 0.39, 0.40, and 0.42 ng/dl, as the qualitative test showed negative results.

Conclusion

This study showed that the qualitative kit is highly sensitive and specific at higher values of troponin I, i.e., ≥ 0.5 ng/dl. The qualitative test could be very beneficial in cost and time savings for the non-conclusive patients, like NSTEMI and ACS in the ED, and patients coming to the outreach chest pain centres where laboratory services are not adequate and whose Trop I values are not very close to the minimum cutoff values.

## Introduction

Cardiovascular diseases (CVD) along with ischemic heart disease (IHD) and stroke are the world’s leading cause of deaths, claiming 17.1 million lives a year [1]. The developing countries contribute a greater share to the global burden of CVD than the developed countries, and it has been estimated that it will claim 7.8 million annual deaths by 2020 [2-3]. CVD just about claims as many lives as cancer, accidents and diabetes mellitus (DM) combined [4].

Cardiovascular disease is accountable for 10% of disability-adjusted life years (DALYs) lost in low and middle-income group countries and 18% in high-income group countries [5]. In South Asia, the expected increase in coronary artery disease (CAD) is likely to be beyond any other region worldwide [6]. CVD alone claims around 20 million annual deaths in Southeast Asia, around 70% more than for the developed world [7].

Pakistan, India, Nepal and Bangladesh together make up what we call the Indian subcontinent. This region is home to 20% of the world’s population and also shares one of the most substantial CVD burdens. [8] Pakistan is a country with a population of 175 million; the incidence of CAD in Pakistan is alarmingly high, with one in five middle-aged adults inflicted with the disease [9-10].

In myocardial infarction (MI), the blood vessel that supplies the heart gets blocked, the area of the heart that does not receive the blood supply starts to die, and if therapy is not started on time, the heart muscle dies. Thus, the time that passes before the patient receives treatment can mean the difference between life and death. It takes 6 hours before myocardial necrosis can be identified by standard macroscopic or microscopic postmortem examination. Complete necrosis of all myocardial cells at risk requires at least 4 to 6 h or longer, depending on the existence of collateral blood flow into the ischemic zone, persistent or intermittent coronary artery occlusion and the sensitivity of the myocytes [11].

Thus, the mainstay of treatment lies in early diagnoses of MI so that treatment can be started at the earliest. The cardiac markers are namely CK-MB, troponin I (cTnI) and troponin T (cTnT). Troponin is a muscle protein which along with calcium ions and tropomyosin allows muscular contraction. The troponins are found both in the cardiac and the skeletal muscles, but cTnT is used in detecting MI rather than CK-MB because it stays in the blood longer and is cardiac-specific. The appearance of these cardiac proteins in blood is thus the most specific and sensitive indicator of acute myocardial cell necrosis [12].

The above cardiac markers can be detected in the lab or with handheld gadgets on the bedside, thus distinguishing patients with non-lethal chest pain and patients presenting with acute coronary syndrome (ACS) [13-16].

According to the American College of Cardiology (ACC) guidelines for non-ST-elevation myocardial infarction (NSTEMI), only one troponin level has to be elevated above the cutoff value to diagnose acute MI [17]. The values start to elevate within two to four hours, and a peak is achieved at 10 to 24 hours. Values then return to normal within 5 to 10 days.

Two methods are employed to detect the cTnI and cTnT levels in the blood: the rapid bedside assay (qualitative test) which takes less than 20 minutes reporting positive and negative and is most commonly employed in the emergency department (ED) and the conventional quantitative test which takes 45-90 minutes but provides almost correct diagnoses of MI [18]. It has been found that the rapid bedside test of cTnT provides an almost immediate diagnosis of MI and the time delay from the onset of symptoms to a positive cTnT result was almost equal for the rapid assay and the enzyme-linked immunosorbent assay (ELISA) method (quantitative test) [12].

Thus, the shorter the door-to-needle time (time taken to administer clot-busting medications after the patient has entered the ED), the more efficient the ED is to cope with a heart attack emergency. According to the American Heart Association (AHA) guidelines, the ED acute myocardial infarction (AMI) protocol says that when a patient comes in with the symptoms of chest pain, a clinical exam and a 12-lead ECG should be done within 10 minutes and the door-to-needle time should be less than 30 minutes.

Using the qualitative test, we will not only reach the diagnosis of an MI but will be able to save a precious amount of time in doing so. The result of this test appears as positive or negative in 15 minutes, as opposed to the quantitative lab tests that require 45-90 minutes on average.

## Materials and methods

Study setting

The study was conducted at the Karachi Institute of Heart Diseases (KIHD), a specialized cardiac tertiary care hospital catering to the needs of the people of Karachi.

Study population

The study population included patients admitted to the KIHD with MI. Most of the patients visiting the KIHD belong to the northern part of the metropolis; however, patients from other parts of the city and Sindh also visit the KIHD.

Inclusion criteria

This study included subjects of either sex presenting with acute coronary syndrome(ACS)/non-ST-elevation myocardial infarction (NSTEMI) admitted to the KIHD, who had a stable state of mind and provided consent to participate.

Exclusion criteria

Patients visiting the outpatient department of KIHD, those with ST-elevation MI or confirmed cases of CAD as per the WHO criteria, and patients who did not consent to participate and did not have a stable state of mind were excluded from the study.

Sample size

This study encompassed 200 consecutive patients of ACS/NSTEMI admitted to the KIHD.

Study design

This is a cross-sectional study.

Method(s) of data collection

Diagnosed patients of ACS/NSTEMI (diagnosing criteria ECG) were selected. After obtaining consent, a standardized questionnaire was administered, and then patients underwent the “SD Bioline Troponin I Rapid Test (Manufactured by Standard Diagnostics, Korea)”. The test device is sensitive to humidity, excessive heat, and refrigeration; hence, it is stored at a pharmacy in cold storage. The test should be immediately performed after removing the kit from packaging, and we followed the same protocol before using the troponin I kit. Eighty microliters of whole blood was added into the sample well using a micropipette. The test results were then interpreted after 15 minutes. One sample was simultaneously sent to the hospital laboratory for quantitative test.

Measurement tools

This study included a detailed standardized questionnaire, Bio Line SD Troponin I (qualitative test), and lab test for quantitative analysis (gold standard); the cutoff value for cardiac troponin I (cTnI) was 0.30 ng/dl.

Ethical consideration

Our procedures were followed in accordance with the ethical standards of the responsible committee on human experimentation (Ethics Review Committee, Karachi Medical and Dental College) and with the Helsinki Declaration of 1975. We do not use patients' names, initials, or hospital numbers, in-text and illustrative material. The study was approved by the Ethics Review Committee (Karachi Medical and Dental College). Patient confidentiality was strictly assured according to the international ethical guidelines. All data input forms were assigned serial numbers which are only accessed by authorized personnel. Consents were taken from the patients before enrolling them in the study.

Statistical analysis 

Descriptive statistics were done to represent our data through means, proportion, and frequency. Sensitivity and specificity of the diagnostic kit were compared against the gold standard test, a quantitative test for Troponin I, and the predictive values of our diagnostic test were calculated. Statistical data analysis was carried out using SPSS V.18 (SPSS Inc., Chicago, IL).

## Results

Our results showed that the majority of the study subjects were male (67%) as compared to females (33%) with a mean age of 56.66 years (S.D ±11.18; Table 1).

**Table 1:** Demographics of the study population

S.No.	Variables	
1	Age	56.66 years (±11.18)
2	Gender	Males: 33%
		Females: 67%

The qualitative test showed 128 positives and 72 negative cases, while the quantitative test, considered as the gold standard, showed 125 positive and 75 negative cases. The troponin I qualitative kit showed 98% (97.66%) sensitivity and 100% specificity as compared to the quantitative test with a cutoff of 0.30 ng/ml. The positive predictive value was found out to be 100%, while the negative predictive value was found to be 96 % (Table 2).

**Table 2:** Sensitivity and specificity of troponin I kit The sensitivity of the kit was found to be 97.66 = 98% and the specificity of the kit was 100%. The positive predictive value was 100% and the negative predictive value was 96%. TP, true positive; TN, true negative; FP, false positive; FN, false negative

Troponin I Qualitative	(+)	(-)	Total
(+)	125 (TP)	0 (FP)	125
(-)	3 (FN)	72 (TN)	75
Troponin I Quantitative (Cutoff 0.3 ng/dl)	128	72	200

## Discussion

Our study results showed 125 true positives (patients labelled positive by both qualitative and quantitative analysis) and showed 72 true negatives (i.e. patients labelled negative by both kit and lab reports). Results showed a discrepancy in three tests in which the quantitative test by the laboratory showed troponin I value of 0.39, 0.40, and 0.42 ng/ml, respectively, which were positive as per the lab cut-off criteria. However, the troponin I assay was not able to detect these values. Hence, the kit showed the subject as negative for the cardiac event at 0.39, 0.40, and 0.42 ng/ml, while the quantitative results showed recent cardiac insult at this cut-off value.

We reviewed the patients with unconfirmed MI and no ST elevation on the ECG and diagnosed with acute coronary syndrome. The results show that the troponin I assay shows 98% sensitivity and 100% specificity, i.e. the qualitative kit was not completely successful in identifying the true positive patients. The cut-off value for the quantitative laboratory test was 0.30 ng/ml, i.e., the quantitative test showed 125 positive and 75 negative cases.

One of the studies done at three German study centers - the Johannes Gutenberg University Medical Center in Mainz, the Federal Armed Forces Hospital in Koblenz, and University Hospital Hamburg-Eppendorf in Hamburg showed that testing of patients with chest pain with a single troponin I assay at the time of admission, as compared with a conventional troponin T assay and other markers of myocardial necrosis, substantially improved the accuracy of early diagnosis of MI [19]. Our study showed that the troponin I assay at 0.50 ng/ml cutoff was not sensitive enough to detect all patients with non-ST-segment elevation MI. Therefore, it can be concluded that the qualitative assay could be immensely specific at values above or equal to 0.5 ng/ml.

One of the strengths of our research was that it was carried out under standardized conditions, in a cardiac hospital setup, with the patients recruited from the emergency room who had a high probability of MI. Troponin assays allow earlier detection of MI even, in patients consulting ED quite early, after the onset of symptoms [19-22]. This kit can be beneficial in settings where quantitative assays are either not available or not available all 24 hours. This is true of many public hospitals which may not be cardiac specific. Also with outreach chest pain centres being developed, this kit usage will decrease the demand for laboratory facilities which will be both staff and cost-intensive. This test may also be beneficial when compared with the more time-consuming quantitative laboratory tests. The use of qualitative test(s) may result in fewer hospital admissions, with the potential for savings in healthcare costs and reduced overcrowding in EDs and hospitals [22].

One of the limitations which may be taken into account is the considerably small sample size of 200 patients only, which is due to minimal financial and human resources. The results show that the troponin I kit missed three subjects at 0.39, 0.40, and 0.42 ng/ml, i.e., was unable to detect levels below 0.5 ng/ml. This measurement may have failed to identify patients with “minor” infarctions who had only low-level increases in troponin I.

Moreover, many tertiary care hospitals have opened outreach satellite clinics at remote areas far from the city centre; these clinics at present due to limited funding usually do not have well-equipped laboratory or other associated services available, which are required for the suitable and appropriate emergency treatment of those patients who are brought in with suspicion of MI. In such cases, the use of the qualitative troponin I kit can be justified. This, in turn, simplifies the selection of the patients at these facilities and can reduce the burden of true-negative or false-positive patients at the tertiary care referral hospital. Also, the shipping of blood samples of such patients by a general practitioner or from outreach satellite clinics to the laboratory for diagnostic purposes can be minimized, and ambulance journeys can be time-consuming, and the usage of this qualitative test can result in the reduction of the door-to-needle time.

So, further studies with an adequate sample size may be able to further strengthen our study results and determine whether the early diagnosis of MI facilitates rapid initiation of treatment and thus improves patient outcomes.

## Conclusions

Our study results conclude that the initial use of the sensitive troponin I assay substantially improved the early diagnosis of MI and helped safely rule out or rule in coronary causes of acute chest pain. This study also suggests that troponin I assay has excellent sensitivity and specificity at higher values of troponin I, i.e., ≥0.5 ng/dL. This qualitative test could be beneficial and cost-effective for patients with suspected CAD, coming to outreach chest pain clinics or other primary care health setups, where laboratory services are inadequate. However, as this is a pilot study, further evaluation necessitates a multi-centre study using a larger sample size of various ethnic groups.
